# Anti-Inflammatory Effect of Garcinol Extracted from *Garcinia dulcis* via Modulating NF-κB Signaling Pathway

**DOI:** 10.3390/nu15030575

**Published:** 2023-01-22

**Authors:** Pathanin Chantree, Pongsakorn Martviset, Nattaya Thongsepee, Kant Sangpairoj, Phornphan Sornchuer

**Affiliations:** 1 Department of Preclinical Science, Faculty of Medicine, Thammasat University, Pathumthani 12120, Thailand; 2 Thammasat University Research Unit in Nutraceuticals and Food Safety, Thammasat University, Pathumthani 12120, Thailand; 3 Research Group in Medical Biomolecules, Faculty of Medicine, Thammasat University, Pathumthani 12120, Thailand

**Keywords:** garcinol, *Garcinia dulcis*, inflammation, THP-1, RAW 264.7, NF-κB

## Abstract

*Garcinia* is a significant medicinal plant with many beneficial phytoconstituents, including garcinol. This study investigated the anti-inflammatory effect of garcinol isolated from *Garcinia dulcis* fruit in LPS-activated THP-1 and Raw 264.7 macrophages. The results demonstrated that the low concentration of garcinol did not alter cell viability. Furthermore, co-incubation of garcinol with LPS inhibited the production of pro-inflammatory cytokines, including TNF-α, IL-8, IL-6, IL-1β, and pro-inflammatory mediators, including iNOS and COX-2 at the mRNA and protein expression levels. Garcinol also decreased the secretion of TNF-α, IL-6, IL-1β, PGE2, and NO. Moreover, the anti-inflammatory effects involved an alteration in the NF-κB signaling pathway. Downregulation of pIKKα/β, pIκBα, and pNF-κB was observed, hence reducing the translocation of pNF-κB from the cytosol into the nucleus, which subsequently decreased the production of pro-inflammatory molecules. Therefore, garcinol isolated from *Garcinia dulcis* is a potential candidate as an anti-inflammatory agent for inflammation-related disease treatment.

## 1. Introduction

In general, the immune system has a responsibility for pathogen clearance. To maintain immune homeostasis, the innate immune system provides a defense mechanism that can rapidly respond to eliminate pathogenic microorganisms. Reaching this desired purpose involves the production of many inflammatory cytokines [1,2]. The immune cells, such as macrophages, recognize lipopolysaccharide (LPS) from the cell membrane of Gram-negative bacteria by using Toll-like receptor 4 (TLR4) [3,4]. The binding of LPS with TLR4 on the cell surface of macrophages triggers a classical inflammatory cascade called the NF-κB signaling pathway [5,6]. Activation of the NF-κB signaling pathway leads to the production of inflammation-related molecules, such as pro-inflammatory cytokines, including tumor necrosis factor-alpha (TNF-α), interleukin-1β (IL-1β), and IL-6, and pro-inflammatory mediators including inducible nitric oxide synthase (iNOS) and cyclooxygenase-2 (COX-2), which are involved with the production of nitric oxide (NO) and prostaglandin E2 (PGE2), respectively [7,8,9].

However, even if moderate inflammation is helpful for the elimination of pathogens, the massive response, such as excessive production of inflammatory cytokines, will damage vital organ tissues of the host and may cause eventual death [10]. Therefore, controlling the overexpression of pro-inflammatory cytokines and the involved mediators may afford an achievable therapeutic strategy for inflammatory disease treatments.

**Figure 1:**
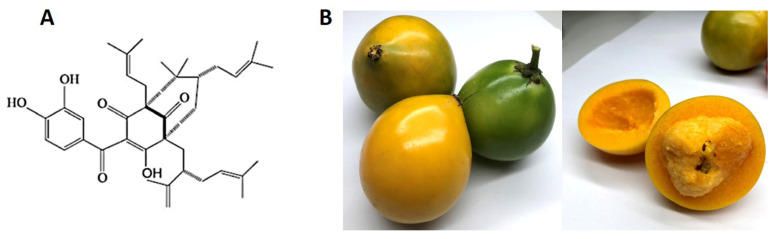
Chemical structure of garcinol (**A**) and *Garcinia dulcis* fruit (**B**).

According to inflammatory treatments, several plant extracts have anti-inflammatory potentials with minimum side effects compared with those of synthetic anti-inflammatory drugs [11]. With more than 200 species, *Garcinia* spp. belongs to the Clusiaceae family and is widely distributed throughout tropical regions. It has been traditionally used in Indian and southeast Asia as medicine by using its leaves, fruits, bark, and roots [12]. *Garcinia* is a significant medicinal plant with many beneficial phytoconstituents, and some species of *Garcinia* are well-studied in research, including *G. mangostana*, *G.cambogia*, *G. pedunculata*, *G. kola*, *G. lanceifolia*, *G. cowa*, and *G. xanthochymus*. Among these *Garcinia* species, *G. indica* (GI) is the most famous specie in several pieces of research about its potential for several disease treatments [13,14]. Garcinol (camboginol) is an isoprenylated benzophenone that is one such instance of a *Garcinia*-derived extract. This compound was first isolated from *Garcinia cambogia* [15]. Its molecular formula is C_38_H_50_O_6,_ with a molecular weight of 602, and its chemical structure was illustrated as shown in Figure 1A [15]. Several studies have revealed that garcinol, especially from the GI fruit, exerts a wide range of biological potentials, including anti-inflammatory [16], antitumor [17], antioxidant [18], antimicrobial [19], and neuroprotective effects [20].

Among the Clusiaceae family, *Garcinia dulcis* (GD) is a less common fruit that is locally known as “Ma-phut” in Thailand or “Mundu” in Malaysia [21,22]. In Thailand, various parts of GD have been used as remedies formulation; the root extract was used as an antipyretic agent; seeds and leaves were used for struma, parotitis, and lymphatitis treatment; the bark of the stem was used as an antiseptic drug, and its fruit juice was used as expectorant for sore throat and cough treatment [23,24,25,26]. Moreover, in vivo study of GD extract demonstrated hypotensive and diuretic effects [27], and GD flower extract was anti-hypertensive in the renovascular hypertension model [28]. Interestingly, among the known *Garcinia*, GD is the only species where the whole fruit is eatable [29] and the GD ripe fruit is a source containing bioactive compounds that might be an excellent agent to treat various chronic diseases, as suggested in previous studies [23,30]. Among these, garcinol from GD was first extracted from its fresh ripe fruits [23]. Furthermore, a recent study demonstrated that garcinol extracted from GD exerted vasorelaxant mechanisms via enhanced endothelial nitric oxide synthase (eNOS) expression [31]. However, a few studies found other biological activities of garcinol extracted from GD.

Recently, we first reported the anti-inflammatory effect of garcinol extracted from GD fruit. We also investigated the related molecular mechanism of its action through the NF-κB signaling pathway in macrophage cell lines.

## 2. Materials and Methods

### 2.1. Garcinol and Cell Culture Treatments

*Garcinia dulcis* (GD) fruits were collected from Songkhla Province, Thailand. Garcinol was isolated from fresh ripe GD fruit as previously described [23] and the extract was kindly provided by Associate Professor Wilawan Mahabusarakam, Department of Chemistry, Faculty of Science, Prince of Songkla University, Hat Yai, Songkhla, Thailand.

THP-1 (human leukemia monocytic cell line) was purchased from ATCC (Manassas, VA, USA). THP-1 cells were cultured in RPMI-1640 (Corning, Manassas, VA, USA) and supplemented with 10% fetal bovine serum (FBS), 100 U/mL Antibiotic-Antimycotic, 10 mM HEPES, 1 mM sodium pyruvate, 2 mM L-glutamine, 2.5 g/L glucose (all of the above were obtained from Gibco, Life Technologies Corporation, Grand Island, NY, USA), and 0.05 mM 2-mercaptoethanol (PanReac AppliChem, Darmstadt, Germany) at 37 °C under 5% CO_2_ in the air-humidified atmosphere. THP-1 cells were differentiated into macrophages by treatment with 100 ng/mL phorbol 12-myristate 13-acetate (PMA) (Sigma Aldrich, Darmstadt, Germany) for 48 h [32]. THP-1 cells were cultured in supplemented media for a further 24 h.

RAW 264.7 (murine macrophage cell line) was purchased from ATCC (Manassas, VA, USA). The cells were maintained in DMEM (Corning, Manassas, VA, USA) and supplemented with 10% FBS and 100 U/mL Antibiotic-Antimycotic (Gibco, Life Technologies Corporation, Grand Island, NY, USA) at 37 °C under 5% CO_2_ in the air-humidified atmosphere.

### 2.2. Cell Viability Assay

Cell viabilities of THP-1 and RAW 264.7 cells were measured by using a 1-(4,5-dimethylthiazol-2-yl)-3,5-diphenylformazan (MTT) assay [33,34]. Briefly, 1 × 10^4^ cells per well were plated into a 96-well microtiter plate using a complete culture medium and incubated for 24 h. After incubation, the culture medium was removed. The medium with garcinol in the presence or absence of 500 ng/mL LPS (Sigma Aldrich, Germany) was added and subsequently incubated for 24 h. After that, 5 mg/mL of MTT solution (Sigma, St. Loius, MO, USA) was added to each well and subsequently cultured at 37 °C for 3 h. After that, the supernatant was replaced with 100 μL DMSO. The absorbance at 490 nm was evaluated in a Multiskan Spectrophotometer (Thermo Scientific, Rockford, IL, USA).

Before assessment of the effects of garcinol, the safety doses were determined by treating the cells with garcinol ranging from 10 to 100 μM without LPS exposure. For determining the effects of garcinol, the co-incubation of LPS and garcinol (10, 20, and 30 µM) or 5 µM of dexamethasone (Sigma Aldrich, Darmstadt, Germany) as a positive control anti-inflammatory agent were performed. In addition, the half-maximal inhibitory concentration (IC_50_) was evaluated as previously described [33,34].

### 2.3. Western Blot Analysis

The protein expression of interested molecules was examined using western blot analysis as previously described [35] with slight modification. In brief, total cellular proteins were extracted using a RIPA cell lysis buffer (Cell Signaling Technology^®^, Danvers, MA, USA) containing protease inhibitors (Merck Millipore Calbiochem™ Protease Inhibitor Cocktail Set III, EDTA-Free, Darmstadt, Germany). A BCA protein assay kit (Pierce™ BCA Protein Assay Kit, Thermo Scientific, Rockford, IL, USA) was used to measure the protein concentrations. Thirty micrograms of total proteins was separated on 12.5% SDS-PAGE and further transferred onto nitrocellulose membranes using the Invitrogen™ Power Blotter System (Invitrogen, Carlsbad, CA., USA). The non-specific bindings were blocked using 5% BSA in pH 7.5 tris-buffered saline (TBS) for 1 h at room temperature. The membranes were subsequently incubated with 1:1000 primary antibodies diluted in 1% BSA in TBS with 0.05% (*v/v*) Tween^®^-20 (TBST) at 4 °C overnight with gentle agitation. The primary antibodies were rabbit anti-β-actin, rabbit anti-IL-1β, rabbit anti-IL-6, rabbit anti-IL-8, rabbit anti-TNF-α, rabbit anti-iNOS, rabbit anti-COX-2, rabbit anti-pNF-κB, rabbit anti-NF-κB, rabbit anti-pIκBα, rabbit anti-IκBα, and rabbit anti-pIKKα/β (Cell Signaling Technology^®^, Danvers, MA, USA). After that, the membranes were washed three times in TBST. After that, the membranes were incubated with 1:20,000 goat anti-rabbit IgG secondary antibody conjugated with AP (Life Technologies, Carlsbad, CA, USA) and diluted in TBST for 1 h at room temperature. The secondary antibody was removed and the membranes were washed three times in TBST. The proteins were visualized using 1-Step™ NBT/BCIP substrate solution (Thermo Scientific, Rockford, IL, USA). The intensity of protein bands was measured by using ImageJ software version 1.53 t. The relative band intensity of the treatment groups was computed using the non-treatment band intensity as a starting point.

### 2.4. Semiquantitative Reverse Transcription Real-Time PCR

The mRNA expressions of TNF-α, iNOS, COX-2, IL-8, IL-6, IL-1β, and a housekeeping control, GAPDH, were determined. First, the total RNA was isolated using a TRIzol reagent (Invitrogen, Carlsbad, CA, USA). Next, SuperScript™ III First-Strand Synthesis System (Thermo Scientific, Carlsbad, CA, USA) was used for first-strand cDNA synthesis. Then, the mRNA expression levels were examined by qRT-PCR using iTaq Universal SYBR Green Supermix (Bio-Rad Laboratories) in a StepOne™ Real-Time PCR System (Applied Biosystems, Foster City, CA, USA). Data were determined using the 2^−∆∆CT^ relative quantification method [36]. The values are presented as the fold change relative to the control. The list of primer sequences used for the qRT-PCR is shown in Supplementary Table S1.

### 2.5. Determination of Cytokine and Mediator Secretion by ELISA Assay

In both cells, the pro-inflammatory cytokines, including PGE2, TNF-α, IL-6, and IL-1β, were determined in the culture media. The media were collected after 24 h treatment of garcinol and used to quantify the level of secretory cytokines using sandwich ELISA Kits (Sigma Aldrich, Merck KGaA, Darmstadt, Germany). Briefly, 100 µL of each sample and standard were added to the wells and further incubated for 2.5 h with gentle shaking at room temperature. Next, the solutions were removed, and each well was washed 4 times with 1X wash solution. Afterward, 100 µL of the corresponding antibody was added to each well and incubated for 1 h with gentle shaking at room temperature. Next, each well was washed, and 100 µL of streptavidin solution was added and incubated for 45 min with gentle shaking at room temperature. Next, the TMB substrate reagent was added and incubated for 30 min with gentle shaking at room temperature in the dark. After that, the stop solution was added, and the absorbance was measured at 450 nm using a Multiskan Spectrophotometer (Thermo Scientific).

### 2.6. Determination of NO and PGE2 Secretion

The Griess test [37] was used to examine the level of NO secretion. After treatment, 50 µL of the supernatants from each culture media were co-incubated with 50 µL of Griess reagent (Sigma Aldrich, St. Louis, MO, USA) for 10 min at room temperature. The absorbance was evaluated at 540 nm using a Multiskan Spectrophotometer (Thermo Scientific). The nitrite concentrations were assessed using a standard sodium nitrite solution curve.

### 2.7. Statistical Analysis

All data were received from three independent experiments performed in triplicate and presented as the mean ± standard deviation (SD). One-way analysis of variance (ANOVA) and Dunnett’s multiple comparison test were used to evaluate the differences between quantitative values. The statistical evaluation and data presentation were provided using GraphPad Prism software version 9.3.1 (GraphPad Software, San Diego, CA, USA). The statistically significant was considered at a *p*-value < 0.05.

## 3. Results

### 3.1. Effect of Garcinol on THP-1 and RAW 264.7 Cell Viability

**Figure 2:**
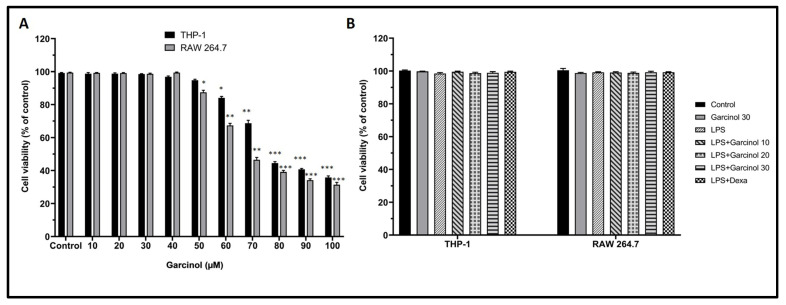
The effect of various concentrations of garcinol on the cell viability of THP-1 cells and RAW264.7 was determined (**A**). The cell viability of another experiment that performed the co-incubation of garcinol or 5 µM of dexamethasone (dexa) with LPS (500 ng/mL) was evaluated (**B**). The results of each group are presented as the relative expression to control. The data are presented as mean ± SD from six replicates examination in three independent experiments. For statistical analyses, One-way ANOVA followed by Dunnett’s test was used. * *p* < 0.05, ** *p* < 0.01, *** *p* < 0.001.

The various concentrations of garcinol ranging from 10 to 100 µM were added to the culture media to optimize the safe dose for further experiments. Each cell viability of RAW264.7 and THP-1 cells was determined by using an MTT assay. After incubation with garcinol for 24 h, the results suggested a decrease in cell viability of RAW264.7 and THP-1 cells in the high concentration of garcinol (Figure 2A). The IC_50_ values of garcinol in RAW264.7 and THP-1 cells were 67.86 ± 1.25 µM and 78.45 ± 2.13 µM, respectively. Hence, the safe concentrations, including 10, 20, and 30 µM of garcinol, were used for both cells in further experiments.

The co-incubation of garcinol (10, 20, and 30 µM) or 5 µM of dexamethasone with LPS revealed no alteration to the cell viability of each experimental group (Figure 2B).

### 3.2. Effect of Garcinol on the Expression of Inflammatory-Related Gene

**Figure 3:**
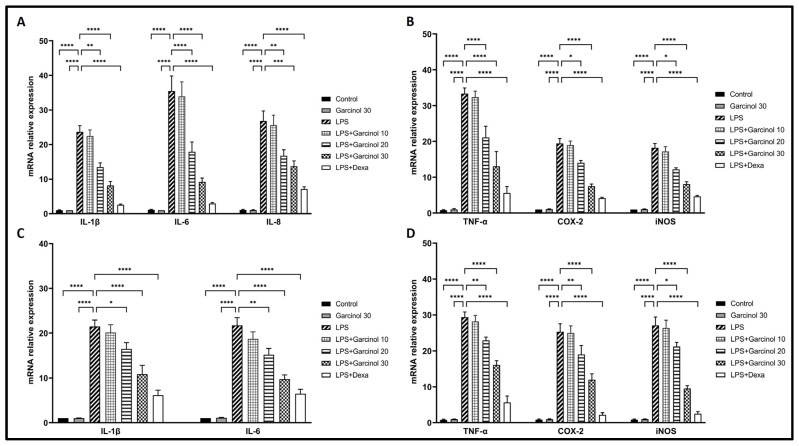
Effects of garcinol (10, 20, and 30 µM) on the mRNA expression level of a pro-inflammatory-related molecule, including TNF-α, COX-2, iNOS, IL-8, IL-6, and IL -1β in THP-1 cells (**A**,**B**) and RAW 264.7 cells (**C**,**D**) activated with LPS 500 ng/mL. 5 µM of dexamethasone (dexa) was used as a positive control. The gene expression was determined by using qRT-PCR. For normalization, the GAPDH gene was used. The results of qRT-PCR of mentioned molecules are presented as a relative fold change of the control. The data are presented in three independent experiments as the mean ± SD of triplicate examination. For statistical analyses, One-way ANOVA and Dunnett’s test were used. * *p* < 0.05, ** *p* < 0.01, *** *p* < 0.001, **** *p* < 0.0001.

The inhibitory effect of garcinol on inflammatory-related gene expression, including TNF-α, iNOS, COX-2, IL-8 IL-6, and IL-1β, was determined. As shown in Figure 3A,B for THP-1 cells and Figure 3C,D for RAW 264.7 cells, the results demonstrated that LPS triggered the massive increase in the mRNA levels of TNF-α (~30–35-fold higher than controls), iNOS (~20–25-fold higher than controls), COX-2 (~20–25-fold higher than controls), IL-8 (~25-fold higher than controls), IL-6 (~20–35-fold higher than controls), and IL-1β (~20–25-fold higher than controls).

Interestingly, co-incubation of garcinol with LPS in THP-1 cells and RAW264.7 cells down-regulated the mRNA expression of inflammatory-related molecules induced by LPS in a dose-dependent manner. However, this counteracted effect was significantly demonstrated in the co-incubation of LPS with only a high dose of garcinol (20 and 30 µM) or 5 µM of dexamethasone with a range of statistical significance when compared to the LPS control group (Figure 3A–D). In the co-incubation of 20 µM garcinol with LPS, the results demonstrated the suppression of increased mRNA levels of TNF-α (~1.4–1.8 fold), iNOS (~1.2–1.7 fold), COX-2 (~1.3–1.4 fold), IL-8 (~1.7 fold), IL-6 (~1.3–2.1 fold), and IL-1β (~1.2–1.3 fold). Additionally, in the co-incubation of 30 µM garcinol with LPS, the results demonstrated the suppression of increased mRNA levels of TNF-α (~1.9–2.9 fold), iNOS (~2.5–3.1 fold), COX-2 (~2.1–3.3 fold), IL-8 (~2.1 fold), IL-6 (~1.9–3.5 fold), and IL-1β (~1.9–2.5 fold). Lastly, in the co-incubation of 5 µM dexamethasone with LPS, the results demonstrated the suppression of increased mRNA levels of TNF-α (~4.9–5.8 fold), iNOS (~3.9–8.3 fold), COX-2 (~5.9–8.3 fold), IL-8 (~3.1 fold), IL-6 (~3.3–11.7 fold), and IL-1β (~3.3–8.3 fold).

### 3.3. Effect of Garcinol on the Expression of Inflammatory-Related Cytokine and Mediators

**Figure 4:**
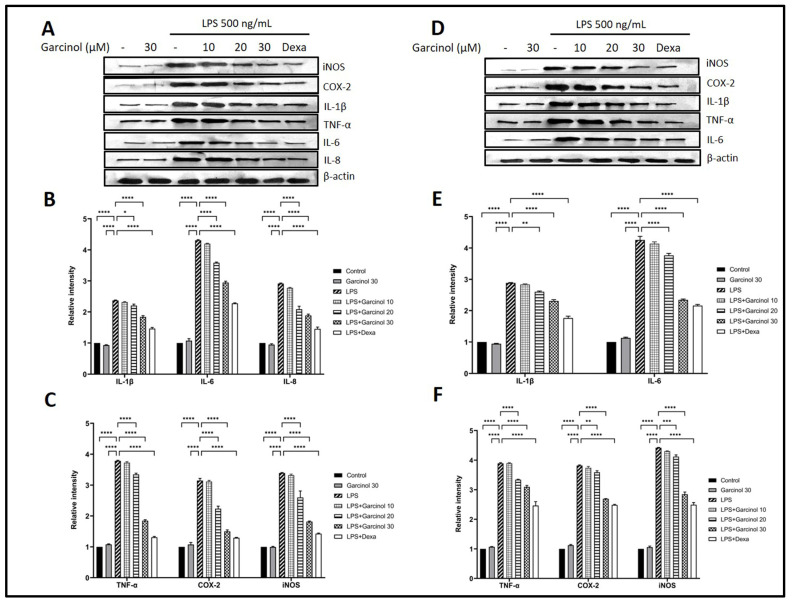
Effects of garcinol (10, 20, and 30 µM) on the protein expression level of a pro-inflammatory-related molecule including TNF-α, iNOS, COX-2, IL-8 IL-6, and IL-1β in THP-1 cells (**A**–**C**) and RAW264.7 cells (**D**–**F**) activated with 500 ng/mL of LPS. Western blot analysis was used to determine the protein expression, and β-actin was used for normalization. The data are presented in the three independent experiments’ mean ± SD of triplicate examination (*n* = 9). For statistical analyses, One-way ANOVA and Dunnett’s test were used. * *p* < 0.05, ** *p* < 0.01, *** *p* < 0.001, **** *p* < 0.0001.

Western blot analysis was used to evaluate whether garcinol disturbs the up-regulation in pro-inflammatory-related molecules after LPS activation. The results suggested the corresponding trend as found in mRNA expression. Following LPS treatment, the relative expression levels of pro-inflammatory molecules, including TNF-α, iNOS, COX-2, IL-8 IL-6, and IL-1β massively increased in THP-1 cells (Figure 4A–C) and RAW264.7 (Figure 4D–F). On the contrary, co-incubation of garcinol with LPS in THP-1 cells and RAW264.7 cells disturbs the increase in protein expression of the mentioned inflammatory-related molecules in a dose-dependent manner with a range of statistical significance. However, in both cells, groups treated with 10 µM of garcinol revealed no significant decrease in these inflammatory-related molecules.

### 3.4. Effect of Garcinol on the Secretion of Inflammatory Cytokines

**Figure 5:**
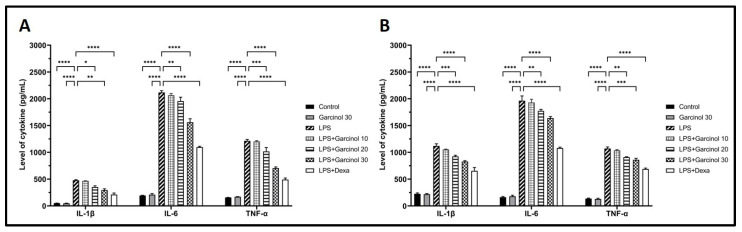
Effects of garcinol (10, 20, and 30 µM) on the secretion of pro-inflammatory cytokines composed of TNF-α, IL-6, and IL-1β were determined in THP-1 cells (**A**) and RAW264.7 cells (**B**) activated with LPS. The supernatants from the culture media of each group were determined for the secretion level of each cytokine using a sandwich ELISA test kit for each cytokine. The data are presented as the mean ± SD of duplicate examination in three independent experiments (*n* = 6). For statistical analyses, One-way ANOVA and Dunnett’s test were used. * *p* < 0.05, ** *p* < 0.01, *** *p* < 0.001, **** *p* < 0.0001.

Using sandwich ELISA kits, the supernatants from the treated THP-1 cells and RAW264.7 cells were used to determine the level of related cytokines. The result suggested that LPS stimulated a vast release of TNF-α, IL-6, and IL-1β compared with the control groups in THP-1 cells (Figure 5A) and RAW264.7 cells (Figure 5B). Meanwhile, in both cells, co-incubation of LPS with garcinol reduced the increase of IL-1β, IL-6, and TNF-α in a dose-dependent manner with a range of statistical significance. However, in both cells, groups treated with 10 µM of garcinol revealed no significant decrease in the releasing level of these inflammatory cytokines compared with the control group (Figure 5A,B)

### 3.5. Effect of Garcinol on the Regulation of Nuclear Factor Kappa B (NF-κB) Signaling Pathway

**Figure 6:**
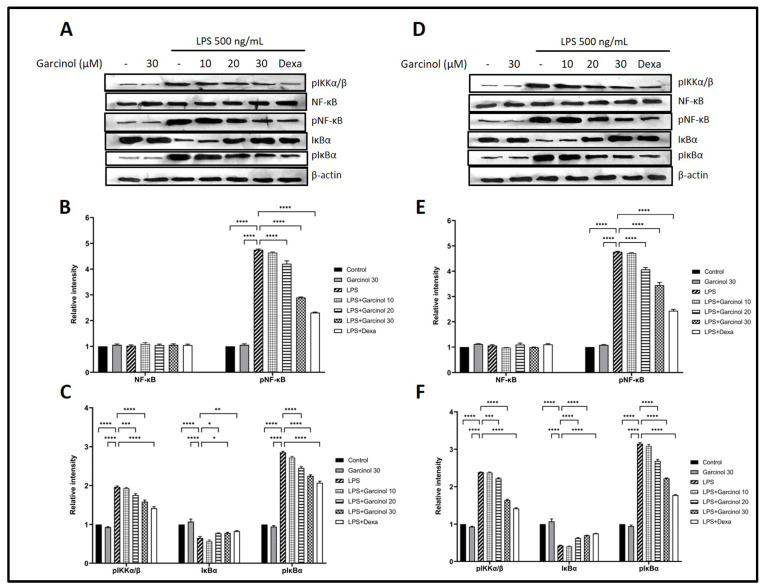
Effects of garcinol (10, 20, and 30 µM) on the expression of related-molecule in the NF-κB signaling pathway in LPS-treated THP-1 cells (**A**–**C**) and RAW264.7 cells (**D**–**F**) were determined by using western blot analysis. β-actin was used for normalization. The data are presented as the mean ± SD of triplicate examination in three independent experiments (*n* = 9). For statistical analyses, One-way ANOVA and Dunnett’s test were used. * *p* < 0.05, ** *p* < 0.01, *** *p* < 0.001, **** *p* < 0.0001.

The effect of garcinol on the modulation of the Nuclear Factor Kappa B (NF-κB) signaling pathway was examined. The protein expression of some related molecules, including pNF-κB, NF-κB, pIκBα, IκBα, and pIKKα/β, were determined by western blot analysis. As shown in Figure 6A–C for THP-1 cells and Figure 6D–F for RAW264.7 cells, treatment with LPS massively increased pIKKα/β, pNF-κB, and pIκBα, meanwhile significantly decreased IκBα. On the other hand, in both cells, the co-incubation with garcinol significantly inhibited the increase of pNF-κB, pIκBα, and pIKKα/β due to LPS activation in a dose-dependent manner with a range of statistical significance (Figure 6). However, in both cells, groups treated with 10 µM of garcinol revealed no significant decrease in these mentioned proteins compared with the control group. Furthermore, there is no significant difference in the expression level of NF-κB among groups in both cells.

### 3.6. Effect of Garcinol on NO and PGE2 Production in LPS-Activated Macrophages

**Figure 7:**
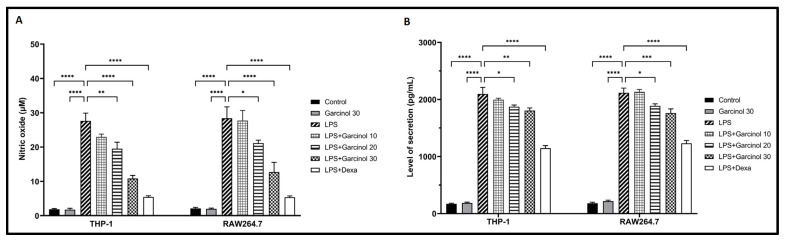
Effects of garcinol (10, 20, and 30 µM) on NO (**A**) and PGE2 (**B**) production were determined in LPS-activated THP-1 cells and RAW264.7 cells. The supernatant from the culture media of each group was determined for the NO and PGE2 secretion level by using a Griess test and ELISA, respectively. The data are presented as the mean ± SD of six replicates, examining three independent experiments. For statistical analyses, One-way ANOVA and Dunnett’s test were used. * *p* < 0.05, ** *p* < 0.01, *** *p* < 0.001, **** *p* < 0.0001.

The supernatant from the culture media of each group was determined for the levels of NO and PGE2 production following LPS activation by using the Griess and ELISA assays, respectively. A massive elevation in NO (Figure 7A) and PGE2 (Figure 7B) production was observed in THP-1 cells and RAW264.7 cells after treatment with LPS. This effect was significantly decreased in the co-incubation groups of LPS with garcinol in a dose-dependent manner with a range of statistical significance. However, in both cells, groups treated with 10 µM of garcinol revealed no significant decrease in the releasing level of NO and PGE2 compared with the control group.

## 4. Discussion

Garcinol, a polyisoprenylated benzophenone derivative, is one of the phytoconstituents extracted from the *Garcinia* fruit [30,38,39,40,41]. Its hydrophobic isoprenyl chain offers the site of biological target attachment. Moreover, the oxidation sites of its chemical structure are composed of a phenyl ring with hydroxyl groups, a double bond of isoprenyl group, and a ketonic group [38,42]. Importantly, garcinol can be considered a prenylated chalcone because its structure is similar to curcumin, a well-known anti-inflammatory and antioxidant agent due to containing two aromatic rings separated by a carbonyl group [43]. Compared with other molecules, garcinol has a molecular structure close to other prenylated compounds, including xanthochymol and cambogin. Xanthochymol is a prenylated chalconoid with a molecular mass of about 602.82 isolated from *G. xanthochymus* [44]. A previous study revealed that xanthochymol has anti-cancer activity [45]. For cambogin, it is the derivative of isoprenylated benzophenone with a molecular mass of about 602.27 found in *G. cambogia* [15,44]. Previous studies suggested that cambogin has biological activities such as anti-cancer and anti-inflammation [46,47]. Furthermore, garcinol also has antioxidant effects. Garcinol revealed the superoxide anion scavenging potency stronger than catechin [18]. In addition, garcinol exhibited free radical 1,1-diphenyl-2-picrylhydrazyl scavenging potency greater than α-tocopherol [48]. Moreover, garcinol was suggested to scavenge hydroxyl radicals, thus suppressing the damage to DNA [18].

Several studies have revealed that garcinol, mostly isolated from the *Garcinia indica* (GI) fruit, exerts many biological potentials, including anti-inflammation [16,17,18,19,20]. In this study, we aimed to study the anti-inflammatory effects of garcinol isolated from a less common *Garcinia* spp., *Garcinia dulcis* (GD)*,* in human and rodent macrophage cell lines. In this study, to eliminate any confounding cytotoxic effects of garcinol, the impact of garcinol on cell viability was examined by MTT assay. After incubation with garcinol for 24 h, the results suggested a decrease in cell viability of RAW264.7 and THP-1 cells in the high concentration of garcinol (Figure 2A). The IC_50_ values of garcinol in RAW264.7 and THP-1 cells were 67.86 ± 1.25 µM and 78.45 ± 2.13 µM, respectively. The cytotoxicity from the high concentration of garcinol probably involved the C8 side chain, one of the main functional groups that give rise to an anti-cancer effect [49]. However, the exact mechanisms for cytotoxicity in macrophages should be further investigated.

In LPS-treated THP-1 cells and RAW264.7 cells, the anti-inflammatory effects of garcinol in mRNA expression levels were determined by qRT-PCR, and related protein expression levels were examined by western blot analysis. The results suggested that garcinol downregulated mRNA and protein expression of TNF-α, COX- 2, iNOS, IL- 8, IL-6, and IL-1β in a concentration-dependent manner (Figure 3 and Figure 4). To evaluate the release level of the significant pro-inflammatory molecules, including IL-1β, IL-6, TNF-α, PGE2, and NO, the supernatants of collected culture media from each group were used to evaluate by using an ELISA test kit and Griess assay. The results revealed the same trend with mRNA and protein expression studies: garcinol reduced the secretion of TNF-α, IL-6, IL-1β (Figure 5), PGE2 (Figure 7B), and NO (Figure 7A). These results implied that garcinol could decrease the production and secretion of major proinflammatory cytokines and mediators. For IL-8, its mRNA and protein expression were only determined in THP-1 cells (Figure 3A and Figure 4A) due to lacking this cytokine in rodents. At the site of inflammation, IL-8 plays as a chemoattractant for T-lymphocytes and neutrophils [50,51,52]. Corresponding to other cytokines in this study, the results revealed that garcinol also decreased IL-8 expression in mRNA and protein levels. Hence, garcinol may inhibit the migration of neutrophils and T-cells, which needs further investigation.

NF-κB is a transcription factor that is involved in inflammation activation. In the resting stage, this molecule typically binds to IκBα, its inhibitor, in the cytoplasm [53]. Following the binding of LPS to the TLR4 complex, the downstream cascades are initiated by multi-subunit IκB kinase (IKK) complex phosphorylation. After that, IκBα is phosphorylated and degraded by the ubiquitin–proteasome system. Because its inhibitor, IκBα, is decreased, free NF-κB translocates from the cytosol into the nucleus and binds to its specific sequence on the enhancer and promoter of target pro-inflammatory cytokine and related mediator genes, resulting in the overproduction of these molecules [53].

Western blot analysis was used to study the expression of related molecules in the NF-κB signaling pathway involved in the downregulation of pro-inflammatory cytokines and mediators induced by garcinol. The co-incubation of LPS with garcinol downregulated the pIKKα/β levels compared with the LPS control group in THP-1 cells (Figure 6A–C) and RAW 264.7 cells (Figure 6D–F). The pIKKs complex is a molecule first produced in the NF-κB signaling pathway that is important for cascade initiation [54]. In this regard, garcinol maybe disturb the NF-κB signaling pathway at the level of the LPS-TRL4 complex formation, which needs to be further elucidated.

**Figure 8:**
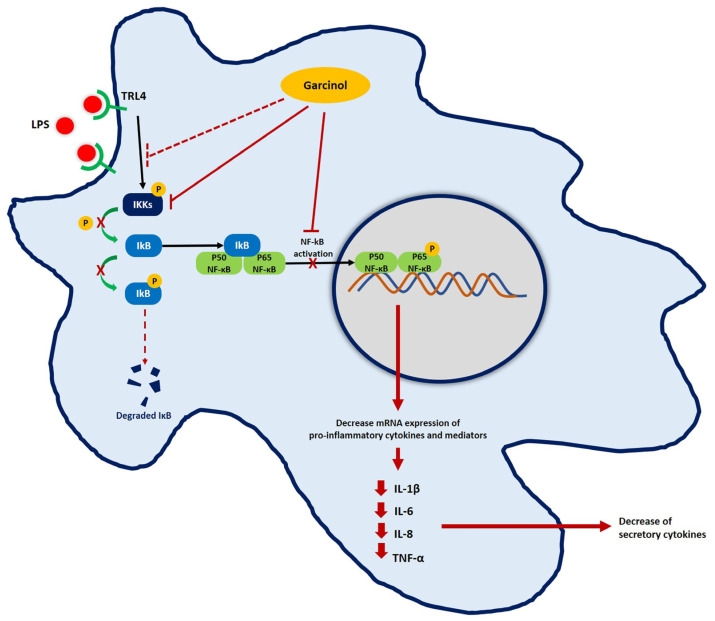
Proposed model for the anti-inflammatory activity of garcinol in macrophages. Black lines represent the standard response for LPS activation; red lines represent garcinol activity found in this present study; and red dashed lines represent the hypothesized activities of garcinol.

The downregulation of pIKKα/β resulted in a decrease of IκBα phosphorylation (Figure 6A,C,D,F); hence, free IκBα was increased, followed by a downregulation in NF-κB-IκBα complex phosphorylation, resulting in a reduction of pNF-κB level (Figure 6A,B,D,E). Therefore, the number of translocations of pNF-κB from the cytosol into the nucleus was decreased. Eventually, the production of pro-inflammatory molecules was decreased in both garcinol-treated cells. In both types of cells, the level of NF-κB was an insignificant difference (Figure 6A,B,D,E). Previous studies reported downregulation, upregulation, or even remaining constant of the expression level of NF-κB after LPS activation. These results suggest that the different concentrations of LPS and incubation time affect the expression level of NF-κB induced by LPS [55,56,57,58]. However, the effect of garcinol on the production of pro-inflammatory molecules while an NF-kB inhibitor is present should also be validated in order to support whether garcinol can suppress the NF-kB signaling pathway. Moreover, the proteasome inhibitor should be used to confirm the effect of garcinol on the proteasomal breakdown pathway [59]. The proposed model of garcinol anti-inflammatory activity based on the results of this study is illustrated in Figure 8.

Previous studies suggested that garcinol isolated from *Garcinia cambogia* inhibited the activation of NF-κB and JAK/STAT-1 signaling pathways in LPS-activated macrophages [60]. Moreover, garcinol isolated from *Garcinia indica* (GI) could decrease inflammation through reduced expression of iNOS and COX-2 [61,62,63]. Furthermore, its effect in altering the binding of LPS to its receptor, including TLR4, was reported. This study suggested its inhibition through decreased IκBα phosphorylation and suppressing p38 MAPK [62]. Additionally, in vitro study of IL-1β-activated chondrocyte suggested the protective effect of garcinol isolated from GI by inhibiting the secretion of pro-inflammatory molecules, including TNF-α, IL-6, iNOS, and COX-2 expression [64]. Hence, based on the previous evidence about the anti-inflammatory effect of garcinol isolated from other *Garcinia* spp., further investigation is needed for other related mechanisms, including JAK-STAT or MAPK signaling pathway. Moreover, the effect on reactive oxygen species production should also be explored in garcinol isolated from GD.

## 5. Conclusions

In a recent study, by using LPS-activated macrophage cell lines as an inflammation activation model, garcinol isolated from *Garcinia dulcis* revealed the anti-inflammatory potentials by its reduction of the pro-inflammatory cytokines and mediators in mRNA and protein expression as well as secretion levels via the modulation of the NF-κB signaling pathway. Therefore, garcinol isolated from *Garcinia dulcis* has the potential as a candidate molecule for the treatment of inflammatory diseases. In the future, the anti-inflammatory activity of garcinol should be further investigated in vivo to develop strategies for controlling an excessive inflammation response, for example, in chronic inflammation-associated diseases, including arthritis, multiple sclerosis, inflammatory bowel disease, and allergy.

## Data Availability

Not applicable.

## Supplementary Materials

The following supporting information can be downloaded at: https://www.mdpi.com/article/10.3390/nu15030575/s1, Table S1. The list of primer sequences used in qRT-PCR.
