# Supplementary material for: Anti-Inflammatory Effect of Garcinol Extracted from Garcinia dulcis via Modulating NF-κB Signaling Pathway
Source: Nutrients. 2023 Jan 22;15(3):575. doi: 10.3390/nu15030575 (PMC9918937; doi:10.3390/nu15030575)
Supplement: Supplementary file 1 [file nutrients-15-00575-s001.zip › nutrients-2170585-supplementary.pdf]

Table S1. The list of primer sequences used in qRT-PCR.

Cell	Gene	Accession no.	Primer sequence from 5′ to 3.′		Product size (bp)
THP-1	TNF-α	NM_000594.4	FW:	GAG GCC AAG CCC TGG TAT G	91
			RV:	CGG GCC GAT TGA TCT CAG C	
	COX-2	NM_000963.40	FW:	CAG CAC TTC ACG CAT CAG TT	128
			RV:	CGC AGT TTA CGC TGT CTA GC	
	iNOS	NM_000625.4	FW:	CGC ATG ACC TTG GTG TTT GG	142
			RV:	CAT AGA CCT TGG GGC TTG CCA	
	IL-1β	NM_000576.3	FW:	CTG AGC TCG CCA GTG AAA TG	202
			RV:	TCC ATG GCC ACA ACA ACT GA	
	IL-6	NM_000600.5	FW:	ACT CAC CTC TTC AGA ACG AAT TG	149
			RV:	CCA TCT TTG GAA GGT TCA GGT TG	
	IL-8	NM_001354840.3	FW:	ACT GAG AGT GAT TGA GAG TGG AC	112
			RV:	AAC CCT CTG CAC CCA GTT TTC	
	GAPDH	NM_001289745.3	FW:	GAG TCA ACG GAT TTG GTC GT	214
			RV:	TGG AAG ATG GTG ATG GGA TT	
RAW 264.7	TNF-α	NM_013693.3	FW:	CCC TCA CAC TCA GAT CAT CTT CT	61
			RV:	GCT ACG ACG TGG GCT ACA G	
	COX-2	NM_011198.4	FW:	TGT GAC TGT ACC CGG ACT GG	233
			RV:	TGC ACA TTG TAA GTA GGT GGA C	
	iNOS	NM_001313922.1	FW:	CCC TTC CGA AGT TTC TGG CAG CAG	497
			RV:	GGC TGT CAG AGC CTC GTG GCT TTG G	
	IL-1β	NM_008361.4	FW:	TGC CAC CCT TTT GAC AGT GAT G	138
			RV:	TGA TGT GCT GCT GCG AGA TT	
	IL-6	NM_031168.2	FW:	CCC CAA TTT CCA ATG CTC TCC	141
			RV:	CGC ACT AGG TTT GCC GAG TA	
	GAPDH	NM_001289726.2	FW:	CGA CTT CAA CAG CGA CAC TCA C	119
			RV:	CCC TGT TGC TGT AGC CAA ATT C	
